# PET-validated EEG-machine learning algorithm predicts brain amyloid pathology in pre-dementia Alzheimer’s disease

**DOI:** 10.1038/s41598-023-36713-0

**Published:** 2023-06-26

**Authors:** Nam Heon Kim, Ukeob Park, Dong Won Yang, Seong Hye Choi, Young Chul Youn, Seung Wan Kang

**Affiliations:** 1 iMediSync Inc, 15F, 411, Teheran-ro, Gangnam-gu, Seoul, Republic of Korea; 2 Department of Neurology, St. Mary’s Hospital, The Catholic University of Korea, Seoul, Republic of Korea; 3 Department of Neurology, Inha University School of Medicine, Incheon, Republic of Korea; 4 Department of Neurology, Chung-Ang University College of Medicine, Seoul, Republic of Korea; 5 National Standard Reference Data Center for Korean EEG, Seoul National University College of Nursing, Seoul, Republic of Korea

**Keywords:** Computational biology and bioinformatics, Neuroscience, Biomarkers

## Abstract

Developing reliable biomarkers is important for screening Alzheimer’s disease (AD) and monitoring its progression. Although EEG is non-invasive direct measurement of brain neural activity and has potentials for various neurologic disorders, vulnerability to noise, difficulty in clinical interpretation and quantification of signal information have limited its clinical application. There have been many research about machine learning (ML) adoption with EEG, but the accuracy of detecting AD is not so high or not validated with Aβ PET scan. We developed EEG-ML algorithm to detect brain Aβ pathology among subjective cognitive decline (SCD) or mild cognitive impairment (MCI) population, and validated it with Aβ PET. 19-channel resting-state EEG and Aβ PET were collected from 311 subjects: 196 SCD(36 Aβ +, 160 Aβ −), 115 MCI(54 Aβ +, 61Aβ −). 235 EEG data were used for training ML, and 76 for validation. EEG features were standardized for age and sex. Multiple important features sets were selected by 6 statistics analysis. Then, we trained 8 multiple machine learning for each important features set. Meanwhile, we conducted paired t-test to find statistically different features between amyloid positive and negative group. The best model showed 90.9% sensitivity, 76.7% specificity and 82.9% accuracy in MCI + SCD (33 Aβ +, 43 Aβ −). Limited to SCD, 92.3% sensitivity, 75.0% specificity, 81.1% accuracy (13 Aβ +, 24 Aβ −). 90% sensitivity, 78.9% specificity and 84.6% accuracy for MCI (20 Aβ +, 19 Aβ −**)**. Similar trends of EEG power have been observed from the group comparison between Aβ + and Aβ −, and between MCI and SCD: enhancement of frontal/ frontotemporal theta; attenuation of mid-beta in centroparietal areas. The present findings suggest that accurate classification for beta-amyloid accumulation in the brain based on QEEG alone could be possible, which implies that QEEG is a promising biomarker for beta-amyloid. Since QEEG is more accessible, cost-effective, and safer than amyloid PET, QEEG-based biomarkers may play an important role in the diagnosis and treatment of AD. We expect specific patterns in QEEG could play an important role to predict future progression of cognitive impairment in the preclinical stage of AD. Further feature engineering and validation with larger dataset is recommended.

## Introduction

Dementia is a fatal disorder marked by a progressive decrease in two or more cognitive abilities, including memory, language, executive and visuospatial functioning, personality, and behavior^1^. Alzheimer's disease (AD) is the leading cause of dementia globally, accounting for almost 70% of cases. AD is characterized by the formation of beta-amyloid (Aβ) plaques and neurofibrillary tangles of hyperphosphorylated tau protein, causing progressive neurodegeneration in specific brain areas^2,3^

AD is difficult to diagnose in its early stages due to the subtle nature of the cognitive loss. Beta-amyloid is a well-characterized diagnostic marker for AD and its aggregation can be used to predict progression from mild cognitive impairment (MCI) to dementia^4^. Yang et al. (2020) reported amnestic SCD with amyloid PET positive showed higher APOE ε4 allele, lower regional brain volume in AD-related areas, lower verbal memory score and higher relative theta/alpha ratio in the frontal area, suggesting a higher possibility of SCD with Aβ + progressing to AD^5^.

In many countries, medications for AD can be prescribed only after the presence of Aβ is confirmed^6,7^. Although cerebrospinal fluid (CSF) and positron emission tomography (PET) biomarkers, combined with relatively new clinical criteria, can help diagnose AD, they are both time-taking and costly^8^. For instance, a recently approved Alzheimer’s drug, Aducanumab, requires repetitive PET and MRI measurement to select a treatment target and monitor side effects. As a result, the demand for more convinient and affordable biomarkers is increasing. Recently, there have been various challenges to identify AD biomarkers utilizing accessible biomarkers. Shen et al. (2020) classified amyloid, tau, or neurodegeneration using a plasma biomarker^9^. Stockmann et al. (2020) designed a panel of structure-based Aβ plasma biomarkers as a prognostic tool for future progression from SCD to MCI or ADD^10^. Buegler et al. (2020) built digital biomarker-based prognostic models which predicted the risk to progress to dementia within 3 years^11^. Cavedoni et al. (2020) reviewed these digital biomarker-based models and recognized their potentials as a new dimensional cognitive-behavioral assessment that can reveal the neural deterioration and impaired cognitive functions, typical of MCI and dementia, even in early stages^12^.

Electroencephalograms (EEG) use multiple scalp electrodes to detect electrical activity in the brain. There have been many trials to make the world a better place, utilizing EEG combined with state-of-the-art techniques, such as BCI^13,14^, Visual Saliency^15^, sleep detection^16^ or fatigue detection^17,18^. In medical fields, previous studies have identified several EEG patterns associated with specific types of brain diseases^19,20^. Several studies have identified distinct EEG patterns that may be used to distinguish AD^21^ or MCI patients^22–25^ from normal subjects, although their statistical power was weak due to limited sample sizes. Lehmann et al. (2007) had some success in classifying AD and normal subjects using an artificial neural network (ANN) with EEG features^26^. Buscema et al. (2007) classified AD and MCI with 92% accuracy using EEG markers as inputs to ANN^27^, whereas Dauwels and colleagues reached 83% and 88% accuracy in classifying pre-dementia and mild AD, respectively^28^. Stromrud et al. (2010) discovered a link between relative theta power and tau/beta-amyloid ratio in CSF^29^. Mander et al. (2015) found correlation between non-REM sleep slow wave activity and beta-amyloid burden^30^. On the other hand, Ho et al. (2020) reported that SCD with Aβ + showed stronger delta and theta waves in resting-state EEG compared to normal, which has similarly been reported for amnestic MCI or AD dementia^31^. Kang et al. found that AD dementia showed an increase in theta and decrease in beta at both scalp and cortical level compared to non-dementia AD subjects and discriminated them accurately with QEEG^32^. Han et al. (2021) also reported similar trend in EEG pattern between encoding and retrieval failure^33^. Moreover, Youn et al. (2020) attempted to find a machine-learning based EEG biomarker for early screening of amnestic MCI, which is the most common phenotype of progressive AD^34^.

More recently, Babiloni and colleagues reported 75.5% accuracy in identifying AD patients and healthy subjects based on EEG features such as power ratios, low-resolution brain electromagnetic tomography (LORETA), and coherences^35^. Meanwhile, Farina et al. (2020) attempted to use EEG combined MMSE score to classify AD, MCI and healthy participants but found no improvement over MMSE score alone^36^. Smailovic et al. (2018) reported that QEEG global field synchronization in alpha and beta range was significantly related to CSF biomarkers^37^. Additionally, Michels et al. (2021) evaluated the relationship between EEG-fMRI and amyloid burden and found that EEG-fMRI signal coupling deteriorated in memory-related regions^38^.

Previous studies have demonstrated significant differences in EEG patterns between AD, MCI, and normal subjects and indicate the possibility for accurate classification according to the progression of cognitive impairment. However, it is a challenge to develop an EEG-based surrogating marker for AD, especially in the preclinical stage. None have succeeded in developing a reliable EEG biomarker for beta-amyloid plaque accumulation and validated it by amyloid PET scan. The current study describes how we adjusted age and sex-related effects on EEG and developed the best machine learning-based EEG biomarker algorithm surrogating brain beta amyloid plaque to achieve high classification accuracy for early detection of preclinical or prodromal Alzheimer’s patients.

## Research method

### Materials

The current study employed a cross-sectional design, collecting EEG data from patients diagnosed with subjective cognitive decline (SCD) or MCI in multicenter cohorts. All enrolled participants were examined using amyloid PET scans. Table 1 presents the distribution of subjects by institution and diagnosis. This study was approved by the Ethics Committees of Korea Dementia Research Center and the Brain Convergence Research Program of the National Research Foundation, Republic of Korea. Written informed consent was obtained from all participants, which have been performed in accordance with the Declaration of Helsinki.

**Table 1:** Number of subjects by institution, diagnosis, age and gender.

	SCD( +)	SCD( −)	MCI( +)	MCI( −)	Total
Institution A	16	69	9	14	108
Institution B	18	77	20	20	135
Institution C	0	4	14	18	36
Institution D	2	10	11	9	32
Age(mean ± sd)	72.0 ± 5.9	71.3 ± 6.9	74.5 ± 6.1	71.5 ± 6.8	71.8 ± 6.7
Gender(M/F)	18/16	94/52	18/11	15/19	145/98
Total	36	160	54	61	311

*SCD(* +*)* SCD (subjective cognitive decline) positive, amyloid PET positive, *SCD( −)* SCD positive, amyloid PET negative, *MCI(* +*)* MCI (mild cognitive impairment) positive, amyloid PET positive, *MCI( −)* MCI negative, amyloid PET negative.

We randomly selected 25% of subjects (N = 76; 13 SCD with Aβ, 24 SCD without Aβ, 20 MCI with Aβ, and 19 MCI without Aβ) to exclude from the model training and use them for subsequent verification. Since the ratio of Aβ + to Aβ − data was markedly skewed, being approximately 1:2.5, the positive data were doubled to balance the training data set. As an augmentation method, each of the Aβ + data were divided into the first half and the second half and treated as two separate sets. For example, if the duration of EEG data is 100 s, we make one dataset from 0 to 50 s, and the other from 50 to 100 s. It is a well-used method for data augmentation in machine learning or deep learning using biological signals^39^.

The SCD inclusion criteria were as follows: (1) persistent subjective complaints of cognitive decline, (2) ≥ 60 years of age, (3) at least 6 years of primary school, (4) a memory test standard score 0 to − 1.5 standard deviations (SD), and the other cognitive tests > − 1.5 SD, and (5) informed consent of the participant. Individuals showing a standard score < − 1.5 SD on any other cognitive test were excluded because of the possibility of MCI. This is a commonly used standard in Korean AD society^40^. We defined MCI in accordance with Petersen’s criteria, which presumes MCI in individuals with objective memory impairment for their age (standard score < − 1.5 SD), but normal performance in activities of daily living (ADL)^41^.

Amyloid PET scans were performed to detect Aβ plaques in the brain. The standardized uptake value ratio (SUVR) was used to quantify cortical Aβ, which was normalized to the cerebellar gray matter. ^18^F-florbetaben PET images were acquired and processed by the precedent procedure. Individual 3D T1-weighted magnetic resonance (MR) images were preprocessed and co-registered into the corresponding PET images. The MR images normalized to a standardized stereotaxic space were divided into three probabilistic tissue maps composed of gray matter, white matter, and CSF. A volume-based template of 90 regions of interest was aligned to the individual MR image. The SUVR was calculated using whole voxels of ^18^F-florbetaben PET images referenced to the cerebellum. The global SUVR was estimated by averaging 90 regional uptake values. All PET images were interpreted by nuclear medicine physicians who were blinded to the neuropsychological tests and classifications and dichotomized the images as amyloid-positive or negative using visual reads. The PET images were interpreted only by readers who successfully completed the electronic training program provided by the manufacturer.

### EEG recording, feature generation and selection

All subjects were instructed to relax with their eyes closed and to refrain from movement and talking. EEG data were recorded (bandpass: 0.1–45.5 Hz; Natus Nicolet EEG v32, Nihon Kohden JE921A and Grass AS40) in the resting-state, eyes-closed condition from 19 scalp electrodes positioned over the whole head according to the International 10–20 System (Fp1, Fp2, F7, F3, Fz, F4, F8, T3, C3, Cz, C4, T4, T5, P3, Pz, P4, T6, O1, O2). A linked-ear reference electrode was noted if present, but not deemed mandatory for the present study, to respect standard internal protocols of the several clinical recording units. In each case, a ground electrode was located between the Afz and Fz electrodes. Electrode impedance was kept below 10 kOhm. All recorded artifact-free EEG data were re-referenced offline to a common average montage to harmonize the EEG data collected using different reference electrodes. All data were digitalized in continuous recording mode (approximately 3 min of EEG; sampling rate: 200 or 250 Hz, to avoid aliasing).

EEG preprocessing was performed to denoise each data to minimize the effects of artifacts. The first stage of EEG preprocessing involved sampling the signals at 250 Hz and filtering them with a bandpass filter in the 1 ~ 45.5 Hz range. After passing the EEG via a notch filter, it was prepared for downstream processing, which included re-referencing (using CAR), bad epoch rejection (using ASR), and removing stationary noise by adaptive mixture independent component analysis (AMICA). Finally, artifacts identified via electromyogram (EMG) and electrooculogram (EOG) were removed to generate QEEG data. All EEG preprocessing processes, sensor-level data, source-level data calculation and extraction were performed using a cloud-based EEG analyzing platform (iSyncBrain^®^, iMediSync, Inc. Korea; https://isyncbrain.com).

Power spectral density of the EEG rhythms was computed using FFT analysis with 0.25 Hz of frequency resolution using iSyncBrain^®^. Then the signal was decomposed into the following frequency bands: delta (1–4 Hz), theta (4–8 Hz), alpha 1 (8–10 Hz), alpha 2 (10–12 Hz), beta 1 (12–15 Hz), beta 2 (15–20 Hz), beta 3 (20–30 Hz), and gamma (30–45 Hz). For each channel and frequency band, the average power during the recoding was calculated and treated as a feature. In addition, we calculated functional connectivity between channels (iCoherence) with the following steps: Coherency between two EEG-channels is a measure of the linear relationship of the two at a specific frequency, following the basic definitions (Nunez et al., 1997). Let x_i_(f) and x_j_(f) be the (complex) Fourier transforms of the time series x̂_i_(f) and x̂_j_(f) of channel i and j; respectively. The cross-spectrum estimate based on the complex valued coefficients for channel pair (*i*,j) is defined as

$${S}_{ij}\left(f\right)= <{x}_{i}\left(f\right){x}_{j}*\left(f\right)>,$$

where x_j_*(f) is a complex conjugate and <  > means expectation value. In practice, the expectation value can only be estimated as an average over a sufficiently large number of epochs. Imaginary coherency is now defined as the normalized cross spectrum:

$$imaginary\, cohernce\,\left(iCoh\right)=im\left(abs\left(\frac{{S}_{ij}\left(f\right)}{{\left({S}_{ii}\left(f\right){S}_{jj}\left(f\right)\right)}^\frac{1}{2}}\right)\right).$$

To adjust the individual variability and age or sex-related physiologic effects on EEGs, all EEG features’ values were standardized into z-score by adopting the age/sex differentiated normative EEG database in iSyncbrain^®^.

To successfully predict morbidity, among the many features associated with Aβ + EEG data, we selected those in which the change was most noticeable in the channel, frequency, or functional connectivity. In many previous machine learning studies, features were selected using various statistical analysis methods to identify those showing significant differences between the target group and the control group. Firstly, we conducted paired t-test to find statistically different features between amyloid positive and negative group. To see the correlation between the two groups, a normality test was performed for each group. Then, Pearson correlation was applied if both groups followed normality. The methods used in the normality test were Shapiro-Wilks test and Kolmogorov–Smirnov test. If either method satisfies normality, we considered that normality was satisfied. If both groups didn’t followed normality, Spearman’s rank correlation test was applied. Additionally, features were identified using 6 methods (T-test, ElasticNet, Whitney-Mann, Random Forest Importance, GBM, XGBoost). MATLAB and Statistics Toolbox R2018b (The MathWorks Inc, Natick, MA, USA) and iSyncBrain^®^ (iMediSync, Inc. Korea; https://isyncbrain.com) were used for statistical analyses. In order to find purely amyloid-specific features and exclude those associated with cognitive impairment, we conducted the same feature selection procedure as previously described to find most significantly distinctive features between SCD and MCI. Then we kept those features from the model-building process so that the final model would consist of pure amyloid-specific features independent of cognitive impairment-related EEG features among MCI or SCD.

### Machine learning modeling and validation

**Figure 1:**
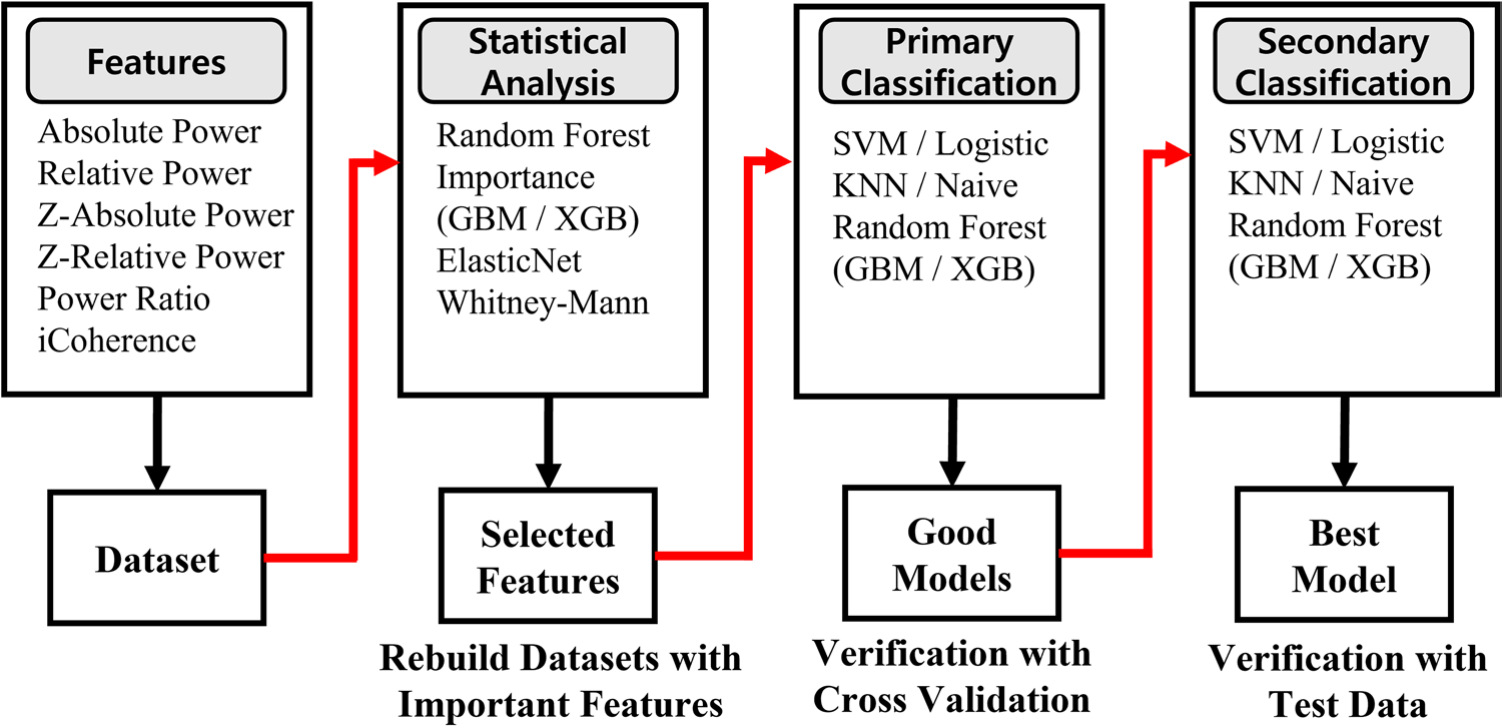
Schematic plot of feature selection with statistical analysis and validation process.

The significant feature sets obtained by the feature selection procedure were then entered into six representative machine learning models, (SVM, Logistic, KNN, Naive Bayes, Random Forest, AdaBoost, GBM and XGBoost), yielding 48 models (6 sets * 8 algorithms). Then the accuracy was calculated by five-layer cross-validation. The feature set that showed more than a predetermined threshold level of accuracy in the above verification was used in the production of this model as an important feature set. MATLAB and Machine Learning Toolbox Rf2018b (The MathWorks Inc, Natick, MA, USA) were used for machine learning. Seventy-six EEGs were used as independent test data set to validate their classification performance. They consisted of 13 SCD Aβ +, 24 SCD Aβ −, 20 MCI Aβ + and 19 MCI Aβ − that had been set aside for validation. The final performance of each model was determined by the results of the test validation. The models with the highest accuracy were selected as the final model candidates. Figure 1 schematically represents the procedure of building classification models from feature generation to feature selection and model validation.

### Deep learning modeling and validation

For comparison, we also conducted deep learning. Feature images were generated based on the standardized image generation protocol of iMediSync, Inc^42^. The standardized spectral powers for each channel were subsequently rearranged and concatenated into a rectangular feature matrix, in accordance with their spatial location on the scalp surface. Application of various feature engineering techniques on the established feature matrix yielded multiple types of feature images. We trained 18-layer ResNet and AlexNet using generated feature images and fivefold cross-validation was applied. MATLAB and Deep Learning Toolbox Rf2018b (The MathWorks Inc, Natick, MA, USA) were used for deep learning. Seventy-six EEGs, the same data for machine learning validation, were used as independent test data set to validate their classification performance.

### Ethics approval and consent to participate

The present study was approved by the Institutional Review Board (IRB) for Human Research at following institutions: Catholic University of Korea, Seoul St. Mary’s Hospital (IRB No: KC18ONDI0394), Inha University Hospital (IRB No: INHAUH2020-10-014), Seoul National University (IRB No: 1711/003-004), Ewha Womans University Mokdong Hospital (IRB no: 2018-08-005) and Ewha Womans University Seoul Hospital (IRB No: 2020-09-006). Written informed consent was obtained from all participants, which have been performed in accordance with the Declaration of Helsinki.

## Results

### Group comparison and modeling process

**Figure 2:**
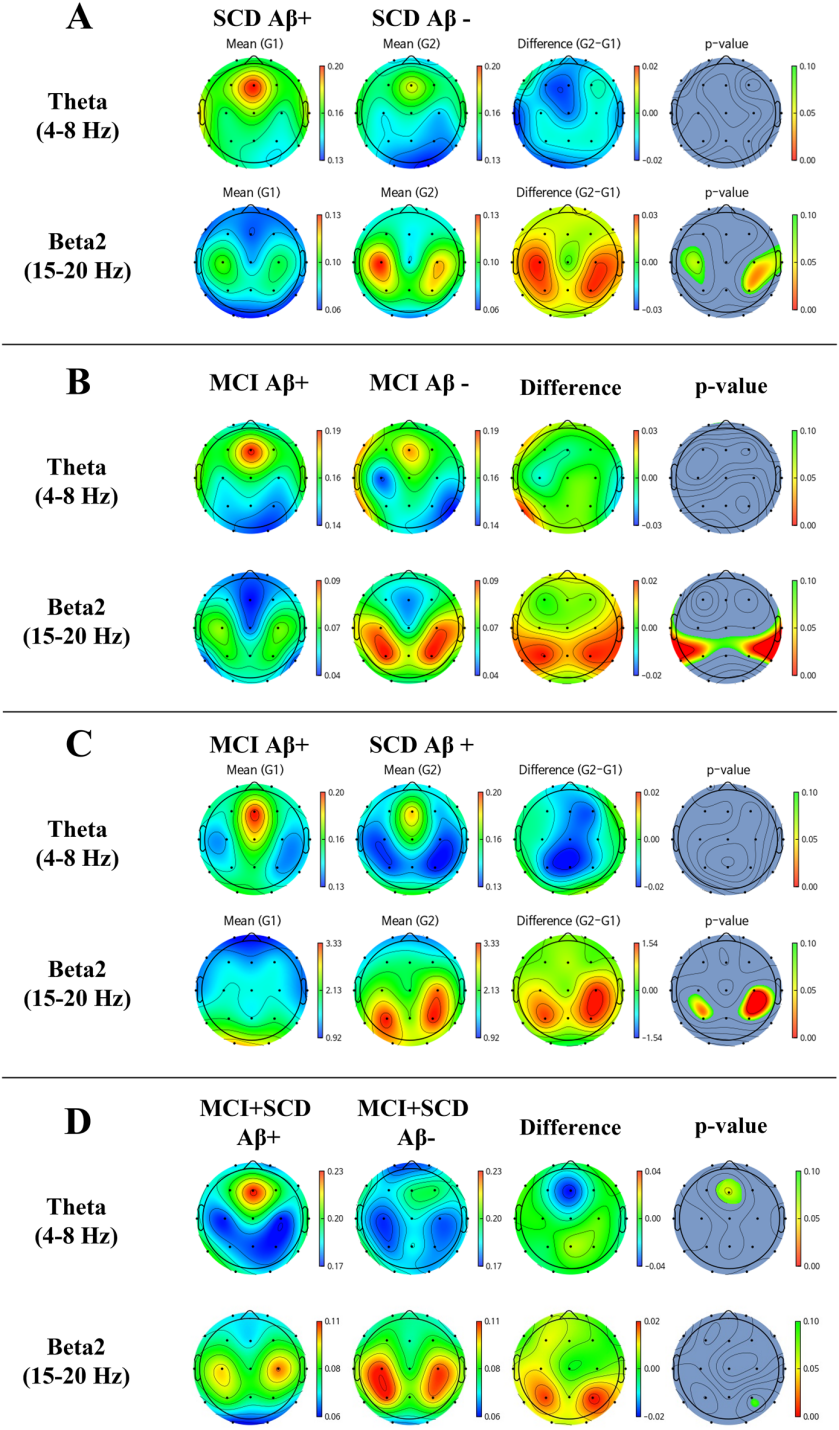
Topomap showing group mean EEG relative power and difference between (**A**) SCD Aβ − and SCD Aβ + , (**B**) MCI Aβ − and MCI Aβ + , (**C**) SCD Aβ + and MCI Aβ + and (**D**) SCD + MCI Aβ − and SCD + MCI Aβ + .

Figure 2 represents the group differences in EEG between amyloid positive and negative were calculated by t-test. We found distinct EEG power patterns between Aβ + and Aβ − in both SCD and MCI. The existence of Aβ plaques presented stronger relative theta power[4–8 Hz] at the middle frontal area and weaker beta2 power[15–20 Hz] at both centroparietal areas. When Aβ + MCI was compared to Aβ + SCD, these patterns became more obvious.

**Figure 3:**
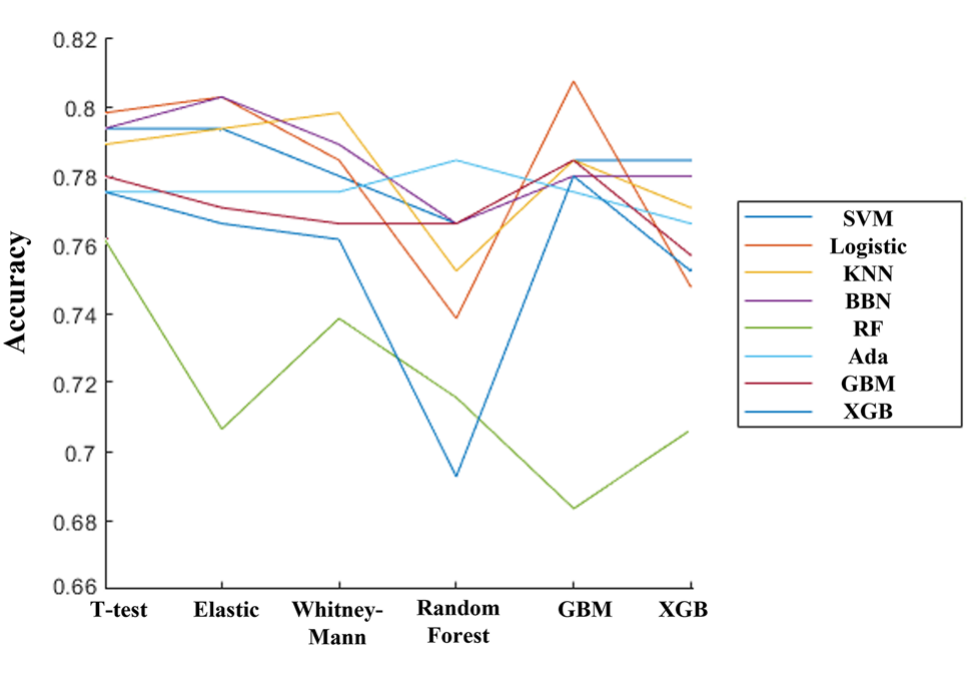
Graph showing accuracy of every feature set and ML algorithm combination. The vertical axis is cross-validation accuracy, and the horizontal axis is statistical analysis for feature selection, each line represents different ML algorithm.

Figure 3 shows the accuracy of models trained with multiple feature sets and multiple ML algorithms. We found various ML algorithms resulting in random classification performances with the use of the same feature set.

**Figure 4:**
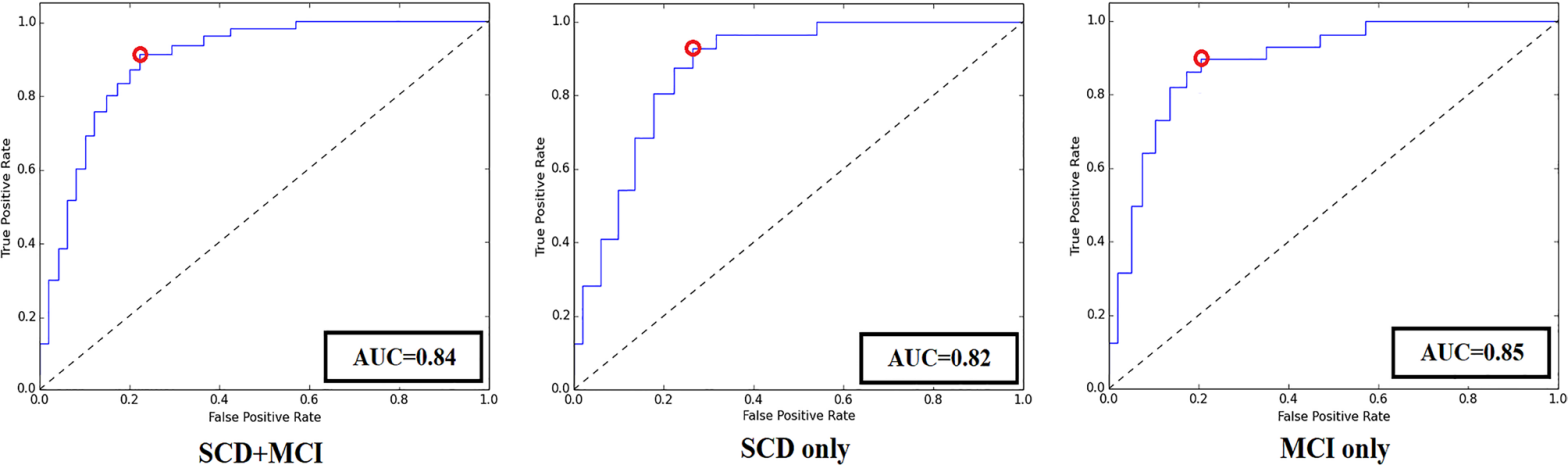
ROC curves of the best classification model. The red circle represents the cut-off point of the model. The AUC was 0.84, 0.82 and 0.85 respectively.

Figure 4 shows the 3rd party data classification accuracy of final model for SCD + MCI group, SCD only group and MCI only group.

### Model validation with 3rd party dataset

**Figure 5:**
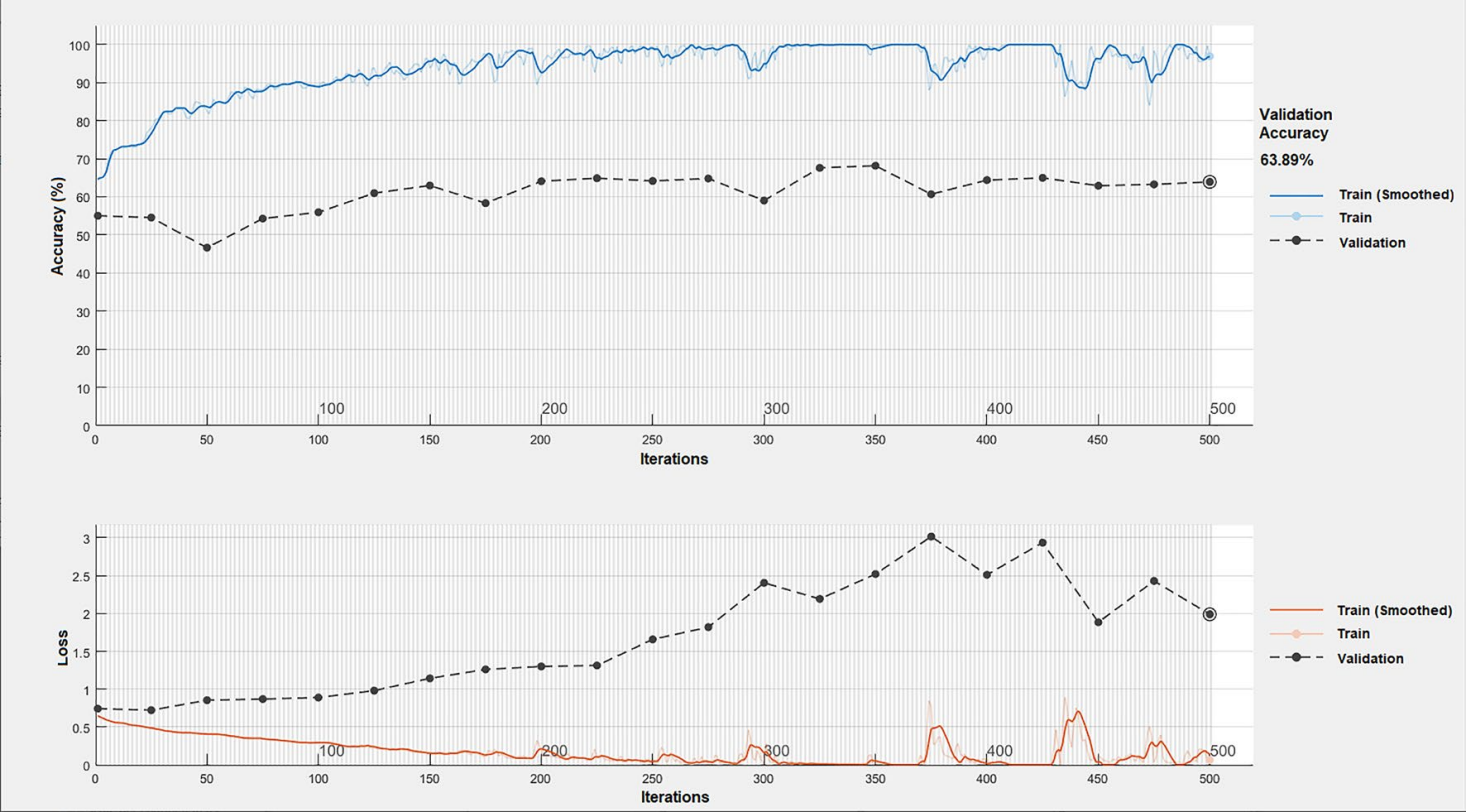
Deep learning curve of best performing model. As the learning continues, train score is getting better while validation score is not getting better, which implies overfitting.

We validated the trained ML model with a total of 76 independent datasets that were not included in the training of ML, and consisted of 13 Aβ + SCD, 24 Aβ − SCD, 20 Aβ + MCI and 19 Aβ − MCI). The best classification model showed 90.9% sensitivity, 76.7% specificity and 82.9% accuracy when it was applied to the total of the SCD + MCI group. When it was applied to the SCD only group, sensitivity was 92.3%, specificity was 75.0 and accuracy was 81.1%. For the MCI only group, 90% sensitivity, 78.9% specificity and 84.6% accuracy were shown. Table 2 presents the confusion matrix. We found overfitting occurs repeatedly during deep learning training, the best validation accuracy was 63.9% in ResNet (Fig. 5). Its 3rd data test showed 60.5% accuracy, 63.6% sensitivity and 58.1 specificity.

**Table 2:** Confusion matrix of classification.

	SCD + MCI		SCD only		MCI only	
	True Aβ +	True Aβ-	True Aβ +	True Aβ −	True Aβ +	True Aβ −
Predicted Aβ +	30	10	12	6	18	4
Predicted Aβ −	3	33	1	18	2	15

## Discussion

We collected resting state eyes-closed EEG from SCI or MCI populations, each of which also underwent amyloid PET analysis for differential diagnosis of AD. The existence of AD among the SCI or MCI population can significantly affect the further progression of cognitive impairment. Early-stage AD, especially when it is at the prodromal stage, is very difficult to detect, and there is no cost-effective biomarker to discriminate the existence of preclinical AD. Although a few previous studies have shown promising results in differentiating normal, MCI or Alzheimer’s dementia by applying various ML algorithms with EEG features, there is no PET-validated study for an EEG-based ML algorithm predicting the existence of brain Aβ+ plaque among SCI or MCI with no less than 80% accuracy, except our previous study^43^. In our previous study, we presented distinct spatial (e.g. posterior cortex) and frequency features (e.g. delta to alpha) of the resting-state QEEG rhythms between Aβ + and Aβ − group, respectively, both in MCI and SCD, and then developed separate ML classification algorithms for Aβ in MCI and SCD individually since we couldn’t get a sufficient number of datasets.

There have been several former trials to confirm the presence of beta-amyloid using resting state EEG alone. However, none of them achieved classification accuracy above 80%. One reason that we could improve the performance is the process of searching best combination of features set and algorithm. There are multiple methods to find good features set, and multiple algorithms to build predictive models. We found the combination of features set and algorithm is important component for performance. As can be seen from Fig. 3, even in a same feature set, performances are fluctuating depending on algorithm adopted. For example, the features set which is obtained by Random Forest showed poor performance with SVM, but showed great performance with XGBoost. In addition, we think traditional machine learning algorithms are powerful enough, but there still remains room for the feature engineering. By trying every combination of features sets and algorithms, we believe we could find the better, hidden model. In previous studies, they could not adjust for the effect of age and sex on the selected EEG features, or effects of cognitive impairment-related EEG changes, which could be the major confounding factors to Aβ-specific EEG features since a relative increase in slow waves and decrease in fast waves are easily observed in normal ageing, neurodegeneration or cognitive impairments. Biases in key features that arise from differences in sex, and age-associated cognitive decline may negatively impact the classification performance of the AI-based algorithms. Furthermore, due to the fact that most patients visiting clinical institutions that suffer from cognitive decline are old-aged populations. In order to overcome such limitations, all selected features were standardized into z-score with the employment of the only age- and sex-differentiated EEG normative database on iSyncBrain^®^^44^. This standardization process could contribute to the improvement of modeling accuracy by the rejection of age- or sex-related EEG changes. The exclusion of EEG features related to cognitive impairment could also be an important factor that contributes to the enhancement of the algorithm’s accuracy, making it more robust and specific to Aβ plaque. In previous studies, various ML algorithms were applied to the same selected features to find the best model. However, there were no studies applying multiple ML algorithms to multiple different feature sets. As a new methodological attempt, we applied multiple ML algorithms exhaustively to multiple different datasets extracted by various statistical techniques. The optimal model was found by verifying every combination of feature sets and ML algorithms. By standardizing age and sex effects based on a normative DB, removing possible confounding features, carefully selecting AD-specific EEG features, combining the best-matched ML model and adjusting the cut-off value for reasonable sensitivity and keeping the overall accuracy, we could get the best results with 90.9% sensitivity, 76.7% specificity and 82.9% accuracy in the validation test with 76 subject datasets consisting of SCD or MCI with or without Aβ pathology. When that ML model was tested for SCD or MCI separately, the validation scores were almost the same: 92.3% sensitivity, 75.0% specificity and 81.1% accuracy were observed for 37 SCD subjects (13 Aβ +, 24 Aβ −). For the MCI only group, 90% sensitivity, 78.9% specificity and 84.6% accuracy were shown for 39 MCI subjects (20 Aβ +, 19 Aβ −). These similar validation results between SCD and MCI means that our ML algorithm is specific for the detection of Aβ pathology regardless of cognitive impairment. In addition, previous studies involved data being collected from a single institution or 2–3 institutions, whilst our study is cohort-based. Hence, we firmly believe that our model is more general, and robust in comparison.

Meanwhile, we couldn’t get a satisfying result from deep learning. We thought the biggest reason for low performance was the lack of data. Another reason was limited computation power. Since we were not equipped with powerful machines for deep learning, we could use relatively shallower networks such as 18-layer ResNet and AlexNet. In addition, in the process of converting EEG, a high-dimensional data, into an image for deep learning, a lot of information such as connectivity and power ratio couldn’t be expressed but only sensor power features.

We conducted several preliminary studies to detect the specific EEG patterns related to AD and observed the characteristics of EEG patterns as being similar to this study where relative theta increased in the frontal or temporal area and decreased in mid-beta [12 ~ 20 Hz] in both centroparietal areas. It was demonstrated that the Aβ + SCD group showed stronger theta activity in the frontotemporal area compared to the Aβ- SCD group, and this pattern was more prominent in the APOE ε4 allele (+) subgroup^31^. A similar patten was enhanced when AD dementia was compared to the non-dementia AD group^32^. Based on these observations, we successfully developed an ML-based classification algorithm to discriminate amnestic MCI from SCD or normal ageing^33^. Various prior studies also repeatedly reported that the enhancement of theta in frontal or frontotemporal and attenuation of mid-beta in centroparietal areas in resting-state EEG were related to MCI or ADD^45–50^. Furthermore, these patterns are not limited to showing the current disease state, but also related to predicting the future progression of cognitive impairment in the preclinical stage of AD. In a prospective study to find predictive factors for the progression of cognitive impairment in amyloid PET-confirmed preclinical AD, the group that progressed from SCD to MCI showed more significant enhancement of theta in midfrontal or temporal areas compared to the stable group. A relative weakening pattern of beta waves in both centroparietal areas was also observed in the progressive group of SCD or MCI^51^.

From the observations in this and other previous studies, we assumed that the enhancement of frontal theta beyond age-related physiological increase could be an EEG biomarker surrogate for neurodegenerative pathology such as Aβ plaque or related cognitive impairment. On the other hand, net-neurocognitive performance isn’t determined only by neurodegenerative pathology, but is also affected by cognitive reserve. Weakened neural compensation is related to attenuated cognitive function, especially in AD^52^. We hypothesize that bilateral enhancement of beta waves in the centroparietal area could be related to neural compensatory activity in ageing or neurodegenerative disease, since this pattern is obvious in preclinical AD, but becomes weak and disappears in AD MCI or ADD. This beta wave range [12–20 Hz] is related to alertness or active information processing and bilateral enhancement of this frequency band isn’t observed in healthy young adults. We will have to conduct further studies to validate the meaning of this phenomenon and relation with neural compensation or cognitive reserve, which could be tentative targets of cognitive enhancement intervention or neuromodulation. Aβ plaque is the main pathology of AD and the diagnostic biomarker of it. However, the quantity of Aβ plaque is weakly correlated with AD-related cognitive impairment, so a decrease in Aβ plaque in amyloid PET can’t predict the prevention of further cognitive impairment or recovery of cognitive function well. Although EEG patterns or ML algorithm for AD are not the direct biological measurement of Aβ plaque, they are a specific endophenotype of underlying Aβ plaque, and could be a surrogate marker for AD-related cognitive functional changes. There have been many efforts to find easily accessible, cost-effective, and noninvasive biomarkers for AD. Compared to various peripheral tentative biomarkers, EEG is the only direct, noninvasive measure of the brain’s real-time neuroelectric activities closely synchronized to emotional, cognitive and behavioral status, or functional brain activities. Advances in computational neuroscience, AI/ML technology and cloud computing have uncovered a large amount of previously invisible information in EEG data and improved its usability in clinical situations.

To summarize the novelty and advantages of our study, although there have been various EEG-based prediction models for Alzheimer’s disease, our study utilized 300 people’s data with amyloid PET results as ground truth in the training of AI models for the first time. The EEG data utilized in our study have been obtained through collaboration of several clinical institutions and universities, allowing the model to be more general and stable. Moreover, through the application of gender- and age-standardized z-score, our prediction model holds robustness to gender- and age-specific differences. On the other hand, the optimal feature set and algorithm were established through combining multiple statistical techniques and varying algorithms, rather than using a single statistical technique or learning algorithm. We expect our classification model to make significant contributions in categorization of dementia or cognitive impairments through predicting amyloid PET results using EEG data. In particular, our model aids segmenting various dementia pathologies and understanding the trends in neurophysiological signal-based longitudinal studies.

Our research suggests that EEG can be useful in early screening of AD and its progression prior to undertaking complex and expensive AD diagnosis procedures, which also contributes to reducing the cost burden on modern society. Our model particularly aids in early-screening for the patients that visit primary and secondary medical institutions that lack in-depth examination methods. Given the recent partial approvement of Aducanumab as a therapeutic drug that targets Amyloid plaques, it is crucial to build foundations for future use and improvements. Our study aims to contribute towards this through the presented amyloid prediction model.

## Limitation

The accuracy of AI/ML models can be enhanced by additional training datasets, more specifically selected and processed features, and the application of advanced algorithms. In this study, we tried to recruit enough subjects for the training dataset. However, feature selection and ML modeling was limited to a data-driven approach. Additional training datasets, more robust feature engineering technology and advanced ML algorithms are necessary to upgrade our algorithm. Then the stability of classification should be continuously tested with bigger datasets recorded with different EEG equipment.

Our model is solely based on physiological characteristics, disregarding conventional clinical tests such as MMSE, APoE4 and MRI. Current model is mono modality-based, and its robustness may be improved through developing multi-modality-based models. For instance, heart rate variability (HRV) characteristics that describe the physiological biases in autonomic nervous system can be considered, as well as other clinical metadata.

Moreover, recent studies report that not the entirety of clinically diagnosed Alzheimer’s disease show positive Amyloid PET results^53,54^. Since our study utilized clinically diagnosed Alzheimer’s disease data which also exhibit positive Amyloid PET results, the model may misclassify Alzheimer’s disease data with negative Amyloid PET results and vice versa.

The relationship between midfrontal theta power or bilateral centroparietal beta power and disease progression or cognitive impairment should be further investigated. There is individual variability among the AD population in EEG patterns, progressiveness, and cognitive function, so we have to verify the existence of different EEG endophenotypes and their relationship with progression or drug responsiveness in AD. Explanation of AI/ML is especially important in medicine to understand pathophysiological mechanisms or explore reversible factors that could be tentative intervention targets. We will develop an explainable deep learning algorithm that should be more accurate and have more specific information about regions of interest, spectral power, functional connectivity and temporal dynamics with additional EEG datasets for AD.

## Conclusion

It is expected that AI/ML-empowered digitalized EEG could work as a cost-effective, easily accessible, and repetitively measurable CNS digital biomarker for AD screening, monitoring, especially when it is combined with easy-to-use wearable EEG devices. It could work for companion diagnostics in AD drug development, contributing to saving time and cost in screening feasible participants and finding the best responders to new interventions based on EEG endophenotypes.

In this study, once deep learning techniques was tried, but the results were not good and the reason is considered to be the lack of data and computational power, and the loss of information in the process of image converting. As a result, classic machine learning techniques were adopted. We believe that the significance of this study is that even existing algorithms can improve performance through feature engineering. In the future study, we will gather more data, and at the same time, we will try various techniques for data augmentation, and on the one hand, we will continue feature engineering and fine tuning of machine learning. On the other hand, we will reinforce the machine and apply the latest deep learning with bigger dataset.

Moreover, we would like to confirm if the existence of different EEG endophenotypes and their relationship with progression or drug responsiveness in AD. Accordingly we will design a longitudinal study to trace subject participated on this study.

Meanwhile, we will analyze the repeatedly misclassified data and find if there is any common pattern, which can be lead to a finding of new endophenotype of Alzheimer’s disease. We expect our classification model to make significant contributions in categorization of dementia or cognitive impairments through predicting amyloid PET results using EEG data. In particular, our model aids segmenting various dementia pathologies and understanding the trends in neurophysiological signal-based longitudinal studies^55,56^.

## Data Availability

The datasets generated and/or analyzed during the current study are not publicly available due to data protection regulations, but are accessible at the corresponding author on reasonable request.

## Abbreviations

AD: Alzheimer’s disease
Aβ: Beta-amyloid
AMICA: Adaptive mixture independent component analysis
ANN: Artificial neural network
EEG: Electroencephalograms
EMG: Electromyogram
EOG: Electrooculogram
LORETA: Low-resolution brain electromagnetic tomography
MCI: Mild cognitive impairment
ML: Machine learning
MR: Magnetic resonance
SCD: Subjective cognitive decline
SUVR: Standardized uptake value ratio
